# Efficacy of web-assisted self-help for parents of children with ADHD (WASH) – a three-arm randomized trial under field/routine care conditions in Germany

**DOI:** 10.1186/s12888-020-2481-0

**Published:** 2020-02-21

**Authors:** Manfred Döpfner, Laura Wähnke, Marie-Theres Klemp, Judith Mühlenmeister, Stephanie Schürmann, Martin Hellmich, Julia Plück

**Affiliations:** 1 Department of Child and Adolescent Psychiatry, Psychosomatics and Psychotherapy, University of Cologne, Faculty of Medicine and University Hospital Cologne, Cologne, Germany; 2 School for Child and Adolescent Cognitive Behavior Therapy (AKiP), University of Cologne, Faculty of Medicine and University Hospital Cologne, Pohligstr. 9, 50969 Cologne, Germany; 3 Institute of Medical Statistics and Computational Biology (IMSB), Faculty of Medicine and University Hospital Cologne, University of Cologne, Cologne, Germany

**Keywords:** Attention-deficit/hyperactivity disorder, Oppositional defiant disorder, Children, Web-based parent management training self-help intervention, e-health

## Abstract

**Background:**

Current clinical guidelines recommend parent management training (PMT) in the treatment of attention-deficit/hyperactivity disorder (ADHD) and oppositional defiant disorder (ODD). However, (a) a lack of supply and (b) structural barriers to attending and continuing face-to-face PMT restrict the access to this training. The main purpose of this study is to investigate the efficacy of online PMT in decreasing ADHD symptoms and oppositional behavior problems and to evaluate the effects of additional telephone-based support of the parents.

**Methods:**

The target sample size is *n* = 495 children with suspected or even clinical diagnosis of ADHD and current symptoms of ADHD or ODD. The study is based on a randomized three-arm parallel group design, in which the effects of treatment as usual (TAU) are compared to TAU plus web-assisted self-help (TAU+WASH) and to TAU plus web-assisted self-help and telephone-based support (TAU+WASH+SUPPORT).

**Discussion:**

The results will provide important insights into the efficacy of web-assisted self-help for parents of children with ADHD and the additional effects of telephone-based support.

**Trial registration:**

German Clinical Trials Register (DRKS) DRKS00013456. January 3rd 2018.

World Health Organization Trial Registration Data Set: Universal Trial number (UTN) U1111–1205-6181. November 23rd 2017.

## Background

Attention-deficit/hyperactivity disorder (ADHD) is one of the most common mental disorders in childhood and adolescence, with a worldwide prevalence of about 5% [1]. Children with ADHD have an increased risk for oppositional (oppositional defiant disorder, ODD), aggressive and antisocial behavior (conduct disorder, CD) problems, besides other comorbid conditions. There is evidence supporting the efficacy of parent management training (PMT) in the treatment of these behaviors. Meta-analyses found that PMT improves parenting and reduces ADHD symptoms and conduct problems in children with ADHD, as well as other externalizing behavior problems [2, 3]. Moreover, recent guidelines recommend behavior therapy (mainly PMT) as well as pharmacotherapy in children with ADHD [4–6]. Over the last few decades, the prevalence of pharmacotherapy has increased significantly across most countries, including Germany, although a downward trend has also emerged in recent years (e.g. [7]). However, the provision of behavioral interventions in Germany is not guaranteed, with strong regional variations. For example, Gebhardt et al. (2008) [8] examined a sample of children and adolescents with ADHD receiving pharmacotherapy, drawn from a German health insurance company, and found that only 19% had received PMT and 7% had received family therapy.

Media-applied PMT may help to overcome this structural lack of therapeutic supply as well as the structural barriers to attending and continuing face-to-face parent training, such as time and work commitments [9]. Telephone-assisted self-help interventions have been found to address some of these barriers and, furthermore, to be effective in the treatment of externalizing behavior problems [10, 11] and to be superior to treatment as usual [12]. Although studies have evaluated internet-based treatments for patients with different disorders [13], research on internet-based self-help for caregivers of children with ADHD is rare. According to a meta-analysis on self-help approaches for parents of children with externalizing behavior disorders [11], two studies on web-based training [14, 15] found small to medium effects (Cohen’s d = 0.42 to 0.72). A Finnish study on internet-based training with telephone counseling for parents of four-year-olds with externalizing behavior disorders found small effects (d = 0.35) [16]. Comparable effects between self-help programs based on self-help books and internet-based self-help programs were found, for example, in Australia [15] and Germany [17–19]. Moreover, self-help was found to enhance the effect of pharmacotherapy in a group of children with ADHD [10]. Recently, Ghaderi et al. [20] found web-based PMT to be as effective as face-to face PMT in children with ODD/CD (including ADHD).

The primary objective of the current study is to assess the efficacy of a newly developed web-assisted self-help program (WASH) for parents of children with ADHD aged 6–12 years. Specifically, the study assesses the efficacy, in terms of reducing externalizing symptoms (ADHD and ODD), of web-assisted parent self-help (WASH) and of WASH combined with additional telephone support (WASH+SUPPORT) compared to treatment as usual (TAU). *The main hypotheses are that (a) WASH + SUPPORT + TAU is more effective than WASH alone, and (b) WASH + TAU is more effective than TAU*. *Moreover, it is expected that face-to-face behavior therapy (ESCAschool) is more effective than WASH + SUPPORT.*

In addition, the following research questions are addressed: (c) acceptance and utilization of WASH compared to WASH+SUPPORT, (d) satisfaction of parents and referring health care providers (HCP, i.e. pediatricians or child and adolescent psychiatrists in their own registered practices) with this intervention, (e) effects of WASH and WASH+SUPPORT on comorbid mental disorders, psychosocial impairment and quality of life of the children as well as on parenting and parental stress, and (f) effects on the utilization of health care services. Furthermore, differential effects (moderators) and mediators of treatment outcome (e.g. sociodemographic variables, parenting and parental stress) are investigated.

## Methods/design

### Participants

Nationwide, all pediatricians as well as child and adolescent psychiatrists working in their own registered practices (*n* = 5015) are contacted, informed about the project and asked to refer patients to the study. Referral by HCPs takes place between December 2017 and February 2020.

To be registered, families have to meet the following inclusion criteria (as rated by the referring HCP): (i) child age 6;0 to 12;11 years; (ii) a (suspected) diagnosis of ADHD; (iii) current symptoms of ADHD or ODD; and (iv) sufficient German language skills of parents (main caregiver with a parenting role for the registered child). Exclusion criteria are: (i) diagnosis of mental retardation or autism spectrum disorder and (ii) indication for inpatient treatment. Caregivers will give written consent (see Appendices) to their HCP to provide personal information to be contacted by the research team.

**Fig. 1:**
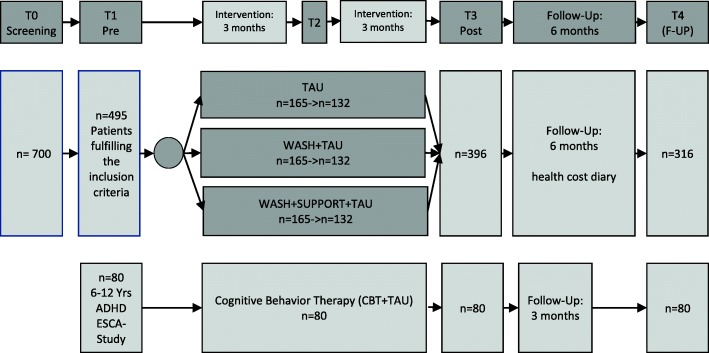
Study design of the ADHD-WASH trial

Participants registered this way (T0, see Fig. 1) receive detailed information about the project (e.g. the technical requirements). After the caregiver’s decision to provide written consent (see Appendices), the inclusion criteria concerning the child’s ADHD or ODD symptoms are checked within the first diagnostic interview (T1). Efforts will also be made to assess patients who drop out of the study.

### Study design

The trial design is described in Fig. 1. The different conditions: web-assisted parent self-help (WASH), WASH plus additional telephone support (WASH+SUPPORT) and treatment as usual (TAU) are compared in a randomized controlled three-arm parallel group design. Approximately *n* = 700 patients are screened for inclusion and exclusion criteria by their HCPs nationwide (T0). If the inclusion criteria are fulfilled within the pre-assessment (T1, *n* = 495), participating families are randomized to one of the three conditions and receive an E-mail with the randomization result as soon as T1 is completed, including the link to the intervention website (WASH and WASH+SUPPORT). Their personal login information is sent separately. If participants have not logged into the website within 5 days, they receive another E-mail, reminding them to use the website and providing the login information again. Participants randomized to the WASH+SUPPORT group are informed about the procedure during an initial telephone call (or E-mail) and receive further telephone assistance (six calls in the first 3 months after completion of T1). An interim assessment (T2) takes place 3 months after the completion of T1, but not before the SUPPORT calls are completed. This makes it possible to record the course of the effects more accurately and to analyze additional effects of the duration of the intervention. The post-assessment (T3, *n* = 396) takes place after a total of 6 months. In all groups, follow-up (T4, *n* = 316) is performed 6 months after completion of the post-assessment. During follow-up (T3 to T4), a health cost diary is completed. Participating caregivers are asked to note every cost (i.e. money, time, additional costs) in a paper-and-pencil protocol. These data are collected during regular telephone calls by research assistants and are transferred into an online survey form.

Additionally, another non-randomized control group of *n* = 80 children aged 6 to 12 years with an ADHD diagnosis is enlisted from a currently running project, ESCA-school [21]. ESCA-school is part of the research network Evidence-based, Stepped Care of ADHD along the life span (ESCA-life) funded by the German Ministry of Education and Research. Participants receive 20 sessions of face-to-face behavior therapy for a period of 6 months. The project has been operating nationwide since January 2016.

### Intervention

The web-assisted parent self-help (https://adhs.aok.de) [22] addresses caregivers of children with ADHD symptoms or oppositional behavior problems. WASH is based on an empirically evaluated treatment program for children with ADHD symptoms and oppositional problem behavior [23] and on a self-help book for parents [19, 24]. This concept has been proven to be effective for families of children with ADHD or other externalizing behavior problems in several studies, both in the form of face-to-face intervention [25] and as telephone-based self-help interventions [19, 26–30].

**Fig. 2:**
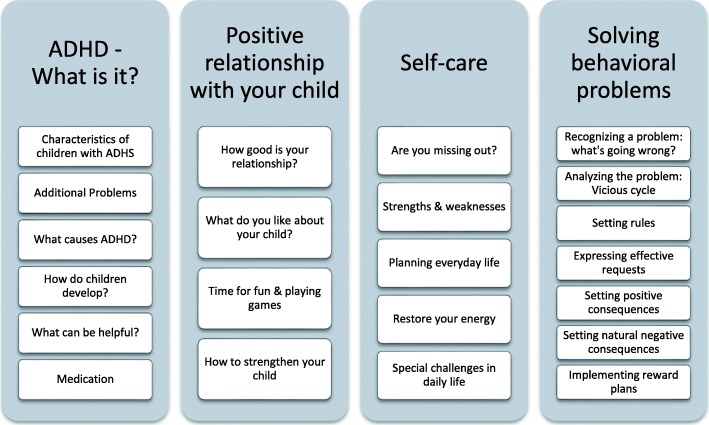
Content and Modules of the ADHD-WASH Program

The structure of the online intervention is shown in Fig. 2. In a matrix design, users are free to take an interest-based approach through the available content of the program. The intervention comprises the following four modules: (1) Psychoeducation (“ADHD – What is it?”), (2) Positive relationship with your child (3) Self-care, (4) Solving behavioral problems. In the fourth module, seven typically problematic situations in the family are presented and users are guided through video-supported examples in seven stages, step by step, by analyzing their own situation with their child and presenting effective methods of change. Caregivers are instructed to use these interventions in everyday life and to document their success within the website. The program consists of tailored (i) text modules, depending on previous (ii) interactive tasks in which users have to assess, for example, their relationship with the child or their own ability to consistently express their individual family rules. Furthermore, (iii) videos and audio recordings are used to make the content easily accessible and to present examples of best practice. Documentation boxes and a personal area, “My Space”, (iv) help to sum up individually relevant aspects and to simplify the adaptation of the WASH recommendations to individual situations. “My Space” serves the purpose of transferring the online information to everyday life (i.e. printable PDF documents analogous to the online tasks).

In the WASH plus SUPPORT group, a trained advisor assists the caregiver with the ADHD parent training. Telephone calls are arranged approximately every 2 weeks (maximum of six 20-min calls within 3 months), during which the advisor discusses the experiences with the program with the caregiver, solves implementation problems and motivates the caregiver to continue his/her work (improve intervention adherence). An individual list of problems (maximum of two) is rated in every call.

### Strategies to improve study adherence

Multiple strategies are applied to retain the families in the study. Participants are given the offer to have the diagnostic results passed on to their HCP. Following T3, families can request information about additional services available to them within the health care and/ or youth welfare system. Participants in the control group (TAU) are provided with analogue material corresponding to the web-based PMT. As far as possible, personnel continuity is aimed at for all assessments in order to create a comforting atmosphere. Families receive “thank-you” postcards after completing T2 and T3 to foster their willingness to participate in the following assessment points.

### Treatment Fidelity

Treatment fidelity in the study, especially in the WASH+SUPPORT group, is assured by (i) a structured and sound training of advisors (guidelines for SUPPORT), (ii) a structured protocol completed by therapists after each session, and (iii) regular supervision of the advisors by senior supervisors.

### Measurements

**Fig. 3:**
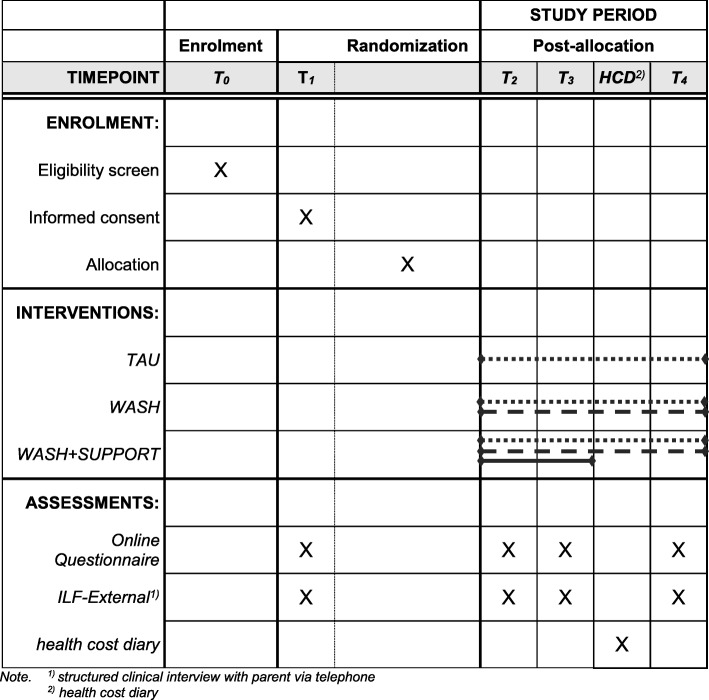
Schedule of enrolment, interventions, and assessments

Data are collected at several *assessment points* before (T1) and after the intervention (T3 & T4) as well as during the *intervention* (T2) (see Fig. 3). Each *assessment point* consists of (i) a clinical interview with the participating caregivers by telephone, as well as (ii) an online survey using the software Lime Survey. At subsequent assessment points (T2 to T4), participants of the intervention groups additionally assess their experience of using the intervention and their satisfaction with it. Further data are generated through (i) assessment by referring HCPs (T1 & T3), (ii) automatically generated utilization data and (iii) caregiver protocols (health cost diary) on the families’ health costs after the intervention.

### Primary and secondary outcomes

#### Primary outcome – blinded clinician

The primary outcome is the externalizing symptomatology in the blinded clinical assessment on German clinical rating scales for ADHD and ODD/CD (*Diagnose Checklist für Aufmerksamkeitsdefizit−/ Hyperaktivitätsstörungen (DCL-ADHS)* and *Diagnose Checklist für Störungen des Sozialverhaltens (DCL-SSV)* [31] based on a structured telephone parent interview (ILF-EXTERNAL) [32]. The blinding is achieved through a second rating of the electronically recorded telephone interview with caregivers. Blinding includes group allocation (three groups) as well as assessment time point (four assessment time points). The externalizing symptoms include ADHD symptoms (18 items) and symptoms of oppositional defiant disorder and conduct disorder (13 items) according to ICD-10 and DSM-5. The original interview guide (ILF-EXTERNAL) was adjusted to the age group, i.e. items for adolescents over 12 years of age were removed.

#### Secondary outcomes – caregiver

The caregiver completes an online survey consisting of the German Symptom Checklist for ADHD (*Fremdbeurteilungsbogen für Aufmerksamkeitsdefizit−/Hyperaktivitätstörungen,* FBB-ADHS) [31], which assesses ADHD symptoms (18 items) and related impairments (five items) as well as the German Symptom Checklist for Oppositional Defiant / Conduct Disorder (*Fremdbeurteilungsbogen für Störungen des Sozialverhaltens,* FBB-SSV) [31], which covers ODD symptoms (eight items), disruptive mood dysregulation disorder (DMDD) symptoms (three items), CD symptoms (six items), characteristics of limited prosocial emotions (11 items), and the child’s impairment due to disruptive behavior problems (five items). The FBB-ADHS and FBB-SSV both consider diagnostic criteria according to the DSM-5 and ICD-10; internal consistency (Cronbach’s Alpha) for the overall symptomology lies at .91, with a range from .68 to .94 for the subscales [31]. A further measure to assess a broad spectrum of emotional and behavioral problems is the German version of the *Child Behavior Checklist for Ages 6–18* (CBCL/6–18R) [33, 34], which has shown satisfactory internal consistencies (a > .70). The child’s quality of life is surveyed through the *KIDSCREEN-10 Index* (10 items) [35], which has demonstrated satisfactory internal consistency (a = .82). Parenting-related measures comprise a German questionnaire for positive and negative parenting behavior (*Fragebogen zum positiven und negativen Erziehungsverhalten,* FPNE) (Cronbach’s Alpha = .91 or .80) to assess functional and dysfunctional parenting practices [36] and the *Depression Anxiety Stress Scale* (DASS; 42 items) to measure parental psychopathology [37], which has shown satisfactory internal consistency (a = .78). Moreover, at T2, T3 and T4, the participants in the intervention groups are asked to report their impression of the intervention through the *User Experience Questionnaire* (UEQ, 26 items, Cronbach’s Alpha ranges from a = .73 to a = .89) [38] as well as their satisfaction with the program using the adapted *Client Satisfaction Questionnaire* (CSQ, 21 items) [39]. Participants who report not having used the program receive questions regarding their non-adherence.

#### Other outcomes

During the use of the intervention, log files from the content management system TYPO3 track the utilization of WASH (i.e. duration and frequency of use of the individual modules). Variables such as first login, last login, number of logins, total login time (in hours), time between logins, number of videos started, number of videos completed, recorded progress etc. are extracted from the system for each user (WASH and WASH+SUPPORT) at T2, T3 and T4. Furthermore, a protocol of all SUPPORT calls (adherence, content of the call, structure of the call etc.) is completed by the advisor after each call (WASH+SUPPORT). Participants in the intervention groups (WASH and WASH+SUPPORT) who have not used the intervention are asked about the reasons for their non-adherence.

Caregivers who withdraw from the study early (dropouts) as well as those who miss out an entire assessment point, are asked to report their reasons for withdrawal or non-attendance and whether they are receiving additional/ alternate provisions. Additionally, ADHD and ODD symptomology are assessed globally (five items from the FBB-SCREEN) [31].

To record the demand for and the cost of health services, participants are led through a health cost diary during the follow-up (for 6 months). Using a protocol of possible costs, they document all treatment events and costs possibly associated with the child’s disorder (for health cost diary, see Becker et al. [40]) and for themselves. Through a combination of specifications and free-text options, it is possible to record costs for health insurance companies as well as private costs.

The following further data are collected from the HCPs: Sociodemographic data about the family at T0 and satisfaction with the study/ intervention at T3 per participant, and again after the last patient out at T4 of a participating practice regarding the overall participation from the HCP’s perspective. Additionally, the psychosocial functional impairment of the child is assessed within the clinical interview with caregivers and directly in the online questionnaire (FBB-ADHS). The satisfaction of referring HCPs is surveyed with a satisfaction questionnaire for medical doctors [41].

### Planned sample size and power calculations

It is assumed that a sample of *n* = 700 patients will be recruited for the initial screening (T0). Based on previous comparable projects, 70% of those in the initial screening are expected to meet the inclusion criteria (T1), so that about *n* = 495 patients will be included and randomized. In online-based self-help studies [15, 16], effect sizes are in the lower to medium range (mostly 0.30 ≤ d ≤ 0.70). Telephone-assisted self-help for parents on the basis of booklets has been found to have moderate effects (0.33 ≤ d ≤ 0.59) [10]. For a comparison of two active conditions (WASH vs. WASH+SUPPORT), rather low to most moderate effects are expected (d = 0.30). Therefore, in this RCT study, the aim is to randomize *n* = 132 (ANCOVA, α = 0.05 two-sided, β = 0.20, correlation pre-post 0.5, 29) [42] participants per group. To compensate for drop-out and cluster effects, an additional 20%, i.e. *n* = 495 (≈3 * 132 * 120%) are included (intention-to-treat set).

#### Strategies to achieve the target sample size

In order to achieve adequate participant enrolment, different strategies are applied: extension of recruiting HCPs (additional inclusion of child and adolescent psychiatrists, direct recruitment via telephone, events, websites), supporting HCPs in enrolment by advertising in different contexts (events, magazines, posters in waiting rooms addressing caregivers directly).

### Statistical analysis

The primary analysis is derived from the intention-to-treat principle, i.e. all randomized patients are analyzed according to their assigned arm, irrespective of whether they refused or dropped out of this treatment or whether other protocol violations occurred.

Linear mixed models for repeated measures (MMRMs) with the fixed effects baseline, group, time, group*time as well as age, sex and symptom severity (at baseline) are fitted (with heterogeneous first-order autoregressive (ARH1) structured variance-covariance matrix over time). The intervention groups are compared by contrasts of marginal means, where the familywise (type I) error is controlled by sequentially conditional rejection (i.e. closed testing). Only patients without valid baseline values are excluded from this analysis. Cluster effects (due to practice, health care provider - HCP) are explored. The influence of missing values is investigated by multiple imputation, particularly those representing missing-not-at-random (MNAR) patterns, where imputation datasets are post-processed by multiplication with factors and addition of offsets (tipping point analysis). The analysis of the participants observed and treated essentially according to the examination plan (per protocol set) is secondary.

The WASH+SUPPORT group and the ESCA-school control group are made comparable to each other (quasi-randomization) by the use of propensity score methods (e.g. by considering age, gender and symptom severity) and differences are analyzed accordingly. The outcome measures of the two studies coincide (symptoms, functional impairment and quality of life).

The moderator analyses refer to characteristics of the parents (e.g. level of education, socioeconomic status, age, psychological stress), characteristics of the family (e.g. family burden, family status) and characteristics of the child (e.g. age, gender, severity of ADHD symptoms, comorbid symptoms). As potential mediators for the effect of the ADHD parent training on the symptoms of the child, the intensity of use of the intervention and the change in parenting behavior are investigated.

### Patient registration and randomization

Patients have to be registered through an HCP nationwide. Participants are assigned to one of three conditions (directly after completion of T1 and after providing informed consent) by a 24/7 Internet service (ALEA, FormsVision BV, Abcoude, NL) that considers (i) gender (female/male) (ii) age (6–9 years or 10–12 years), and (iii) regional factor (rural/urban). The result of the randomization (including further instructions) is sent to the family by e-mail. The randomization is carried out by a member of the research project using the Internet service maintained by the Institute of Medical Statistics and Bioinformatics (IMSB), University of Cologne.

### Data protection

Data are entered into and processed confidentially within the protected and highly secure IT system of the University Hospital Cologne. Moreover, access to the project database is restricted to authorized personnel of the research group only. Data security is ensured by pseudonymization, i.e. personal data (name, contact details, etc.) are saved separately from the actual scientific data (assessments etc.). These two databases are linked only via a password-protected table of identification numbers (ID).

### Stopping rules

A withdrawal of informed consent of parents/guardians following successful inclusion of a participant/ family (T1 completed) results in an early study termination and is rated as a dropout. If the research team is unable to contact a participating family through all available contact information (telephone, E-mail or post), further attempts are undertaken at each subsequent assessment point. There is no stopping rule in this case. If, during the course of participation in the study, there is a need for inpatient treatment of the child, the caregiver may continue to use the allocated intervention.

### Legal and ethical foundations

Prior to the start of the recruitment, all relevant documents have been submitted to and accepted by the ethics committee of the University of Cologne. All constitutive changes to the study protocol have to be reported. Participants have to be informed about necessary changes in the study conditions. Participants are informed verbally and receive information sheets about the scope and the relevance of the study as well as the manner of data collection and processing. All participants can clarify questions about the study with research staff members. Provision of assent and informed consent by either the parents or guardians is necessary for study participation.

### Risk-benefit considerations

Participation in the study does not interfere with the treatment autonomy of the referring pediatrician. For participating children, there is no limitation on other treatment options or changes during study participation (e.g. pharmacotherapy, psychotherapy).

The efficacy of analogue guided self-help interventions (e.g. booklets) for parents of children with similar behavioral difficulties has been confirmed in several studies. The control group (TAU) receives the analogue self-help material intervention 6 months after the beginning of intervention period. There are no known risks involved in the guided self-help intervention.

Patients might be able to draw direct personal benefit from participation in the study (e.g. through a more intensive support) and future patients might benefit through the provision of an evaluated online self-help intervention, especially in geographical regions with a lack of therapeutic supply.

All risks considered, the benefits of the study exceed any possible risks.

## Discussion

Clinical guidelines recommend parent management training in the treatment of children with ADHD. However, the evidence regarding the efficacy of web-based self-help for parents in the treatment of children and adolescents with ADHD is limited. The project assesses the acceptability and efficacy of web-based self-help with or without additional telephone support and investigates whether web-based self-help can improve the routine treatment of children with ADHD or suspected ADHD. The results of ADHD-WASH will lead to a better understanding of the direct, short-term and long-term success parameters and also of moderators and mediating mechanisms of action. The impact on health services and costs estimated for a period of 6 months after the end of the intervention will be analyzed. The non-randomized comparison with individual face-to-face behavior therapy may provide evidence for the efficacy and cost-effectiveness of this intervention compared to face-to-face therapy.

Moderator analyses can provide information on characteristics of parents, family or child, and on acceptance and use of web-assisted self-help, which affect primary and secondary success parameters. Thus, approaches for a target group-specific intervention of the intervention can be gained. The web-based self-help without further support or advice would be immediately feasible nationwide. If telephone assistance proves to be helpful and cost-effective, it could mitigate the blatant lack of psychotherapeutic care for children with ADHD, which the German health system needs to deal with.

## Data Availability

Data will not be shared for the following reasons: at this stage of the study the data collection is not finished.

## Abbreviations

ADHD: Attention-Deficit/Hyperactivity Disorder
CBCL: Child Behavior Checklist
CSQ: Client Satisfaction Questionnaire
DASS: Depression Anxiety Stress Scale
DCL: *Diagnose Checkliste*, Engl. Diagnosis Checklist
DMDD: Disruptive Mood Dysregulation Disorder
DSM5: Diagnostic and Statistical Manual of Mental Disorders
FBB-ADHS: *Fremdbeurteilungsbogen ADHS*, Engl. External assessment sheet ADHD
FBB-SSV: *Fremdbeurteilungsbogen Störung des Sozialverhaltens*, Engl. External assessment sheet Conduct Disorder
FPNE: *Fragebogen zum positiven und negativen Erziehungsverhalten*, Engl. Questionnaire on positive and negative parenting
ICD-10: International Statistical Classification of Diseases and Related Health Problems
ILF: *Interviewleitfaden*, Engl. Interview guide
IMSIE: *Institut für Medizinische Statistik und Bioinformatik der Universität zu Köln*, Engl. Institute for Medical Statistics and Bioinformatics of the University of Cologne
RCT: Randomized controlled trial
SUPPORT: Telephone support in web-based intervention group
TAU: Treatment as usual
TYPO3: Content Management System TYPO3
UEQ: User Experience Questionnaire
WASH: Web-assisted self-help

## References

[1] Polanczyk G, de Lima MS, Horta BL, Biederman J, Rohde LA (2007). The worldwide prevalence of ADHD: a systematic review and metaregression analysis. Am J Psychiatry.

[2] Daley D, van der Oord S, Ferrin M, Danckaerts M, Doepfner M, Cortese S (2014). Behavioral interventions in attention-deficit/hyperactivity disorder: a meta-analysis of randomized controlled trials across multiple outcome domains. J Am Acad Child Adolesc Psychiatry.

[3] Evans SW, Owens JS, Bunford N (2014). Evidence-based psychosocial treatments for children and adolescents with attention-deficit/hyperactivity disorder. J Clin Child Adolesc Psychol.

[4] Taylor E, Döpfner M, Sergeant J, Asherson P, Banaschewski T, Buitelaar J (2004). European clinical guidelines for hyperkinetic disorder – first upgrade. Eur Child Adolesc Psychiatry.

[5] National Institute for Health and Clinical Excellence (NICE) (2018). Attention deficit hyperactivity disorder: diagnosis and management. NICE guideline (NG87).

[6] Deutsche Gesellschaft für Kinder- und Jugendpsychiatrie Psychosomatik und Psychotherapie (DGKJP). (Hrsg) Leitlinienreport der evidenz- und konsensbasierten Leitlinie (S3) Störungen des Sozialverhaltens: Empfehlungen zur Versorgung und Behandlung. AWMF-Registernummer 028–020. 2018. Available from https://www.awmf.org/uploads/tx_szleitlinien/028-045l_S3_ADHS_2018-06.pdf [cited 2019 15 Oct].

[7] Roick C, Waltersbacher A, Klauber J (2016). Administrative Prävalenz und medikamentöse Behandlung hyperkinetischer Störungen bei Kindern und Jugendlichen in Deutschland 2006 bis 2013. Versorgungs-Report 2015/2016.

[8] Gebhardt B, Finne E, von Rahden O, Kolip P (2008). ADHS bei Kindern und Jugendlichen. Befragungsergebnisse und Auswertungen von Daten der Gmünder ErsatzKasse GEK. Schriftenreihe zur Gesundheitsanalyse, Band 65.

[9] Friars P, Mellor D (2009). Drop-out from parenting training programmes: a retrospective study. J Child Adolesc Ment Health.

[10] Dose C, Hautmann C, Buerger M, Schuermann S, Woitecki K, Doepfner M (2017). Telephone-assisted self-help for parents of children with attention-deficit/hyperactivity disorder who have residual functional impairment despite methylphenidate treatment: a randomized controlled trial. J Child Psychol Psychiatry.

[11] Tarver J, Daley D, Lockwood J, Sayal K (2014). Are self-directed parenting interventions sufficient for externalising behaviour problems in childhood? A systematic review and meta-analysis. Eur Child Adolesc Psychiatry.

[12] McGrath PJ, Lingley-Pottie P, Thurston C, MacLean C, Cunningham C, Waschbusch DA (2011). Telephone-based mental health interventions for child disruptive behavior or anxiety disorders: randomized trials and overall analysis. J Am Acad Child Adolesc Psychiatry.

[13] Hollis C, Falconer CJ, Martin JL, Whittington C, Stockton S, Glazebrook C, Davies EB (2007). Annual research review: digital health interventions for children and young people with mental health problems - a systematic and meta-review. J Child Psychol Psychiatry.

[14] Enebrink P, Högström J, Forster M, Ghaderi A (2012). Internet-based parent management training: a randomized controlled study. Behav Res Ther.

[15] Sanders MR, Dittmann CK, Farruggia SP, Keown LJ (2014). A comparison of online versus workbook delivery of a self-help positive parenting program. J Prim Prev.

[16] Sourander A, McGrath PJ, Ristkari T, Cunningham C, Huttunen J, Lingley-Pottie P, Hinkka-Yli-Salomäki S, Kinnunen M, Vuorio J, Sinokki A, Fossum S, Unruh A (2016). Internet-assisted parent training intervention for disruptive behavior in 4-year-old children: a randomized clinical trial. JAMA Psychiatry.

[17] Döpfner M, Schuermann S (2011). Wackelpeter und Trotzkopf. Hilfen für Eltern bei ADHS-Symptomen, hyperkinetischem und oppositionellem Verhalten.

[18] Ise E, Kierfeld F, Döpfner M (2015). One-year follow-up of guided self-help for parents of preschool children with externalizing behavior. J Prim Prev.

[19] Döpfner M, Liebermann-Jordanidis H, Kinnen C, Hallberg N, Mokros L, Benien N, Mütsch A, Schürmann S, Wolf Metternich-Kaizman T, Hautmann C, Dose C. Long-term effectiveness of guided self-help for parents of children with ADHD in routine care - an observational study. J Atten Disord. 2018 (epub ahead of print). doi:10.1177/1087054718810797. 10.1177/1087054718810797 30449268

[20] Ghaderi A, Kadesjo C, Bjornsdotter A, Enebrink P (2018). Randomized effectiveness trial of the family check-up versus internet-delivered parent training (iComet) for families of children with conduct problems. Sci Rep.

[21] Döpfner M, Hautmann C, Dose C, Banaschewski T, Becker K, Brandeis D, Holtmann M, Jans T, Jenkner C, Millinet S, Renner T, Romanos M, von Wirth E (2017). ESCAschool study: trial protocol of an adaptive treatment approach for school age children with ADHD including two randomized trials. BMC Psychiatry.

[22] Schürmann S, Döpfner M, for AOK-Bundesverband. ADHS-Elterntrainer. 2017; Available from https://adhs.aok.de/ [cited 2019 7 Nov].

[23] Döpfner M, Schürmann S, Frölich J (2019). Therapieprogramm für Kinder mit hyperkinetischem und oppositionellem Problemverhalten.

[24] Döpfner M, Schürmann S (2017). Wackelpeter und Trotzkopf. Hilfen für Eltern bei ADHS-Symptomen, hyperkinetischem und oppositionellem Verhalten (5. ed.).

[25] Döpfner M, Breuer D, Schurmann S, Wolff Metternich T, Rademacher C, Lehmkuhl G (2004). Effectiveness of an adaptive multimodal treatment in children with Attention-Deficit Hyperactivity Disorder - global outcome. Eur Child Adolesc Psychiatry.

[26] Hautmann C, Dose C, Duda-Kirchhof K, Greimel L, Hellmich M, Imort S, Katzmann J, Pinior J, Scholz K, Schürmann S, Wolff Metternich-Kaizman T, Döpfner M (2018). Behavioral versus nonbehavioral guided self-help for parents of children with externalizing disorders in a randomized controlled trial. Behav Ther.

[27] Mokros L, Benien N, Mütsch A, Kinnen C, Schürmann S, Wolf Metternich-Kaizman T, Breuer D, Hautmann C, Ravens-Sieberer U, Klasen F, Döpfner M (2015). Angeleitete Selbsthilfe für Eltern von Kindern mit Aufmerksamkeitsdefizit- / Hyperaktivitätsstörung: Konzept, Inanspruchnahme und Effekte eines bundesweitern Angebotes – eine Beobachtungsstudie. Z Kinder Jug-Psych.

[28] Katzmann J, Hautmann C, Greimel L, Imort S, Pinior J, Scholz K, Döpfner M (2017). Behavioral and non-directive guided self-help for parents of children with externalizing behavior: mediating mechanism in a head-to-head comparison. J Abnorm Child Psychol.

[29] Kierfeld F, Döpfner M (2006). Bibliotherapie als Behandlungsmöglichkeit bei Kindern mit externalen Verhaltensstörungen. Z Kinder Jug-Psych.

[30] Kierfeld F, Ise E, Hanisch C, Görtz-Dorten A, Döpfner M (2013). Effectiveness of telephone-assisted parent-administered behavioural family intervention for preschool children with externalizing problem behaviour: a randomized controlled trial. Eur Child Adolesc Psychiatry.

[31] Döpfner M, Görtz-Dorten A (2017). Diagnostik-System für psychische Störungen nach ICD-10 und DSM-5 für Kinder- und Jugendliche - III.

[32] Görtz-Dorten A, Thöne AK, Döpfner M. Interviewleitfäden zum Diagnostik-System für psychische Störungen für Kinder- und Jugendliche (DISYPS-III-ILF) Bern: Hogrefe; 2020.

[33] Achenbach TM, Rescorla LA (2001). Manual for the ASEBA School-age Forms & Profiles.

[34] Döpfner M, Plück J, Kinnen C (2014). Arbeitsgruppe Deutsche Child Behavior Checklist. Elternfragebogen über das Verhalten von Kindern und Jugendlichen (CBCL/ 6-18R). Deutschsprachige Fassung der Child Behavior Checklist for Ages 6–18 von Thomas M. Achenbach.

[35] KIDSCREEN (2006). Group Europe. The KIDSCREEN questionnaires: quality of. life questionnaires for children and adolescents.

[36] Imort S, Hautmann C, Greimel L, Katzmann J, Pinior J, Scholz K, et al. Der Fragebogen zum positiven und negativen Erziehungsverhalten (FPNE): Eine psychometrische Zwischenanalyse. Poster zum 32 Symposium der Fachgruppe Klinische Psychologie und Psychotherapie der Deutschen Gesellschaft für Psychologie. Braunschweig, Germany: DGPs 2014.

[37] Lovibond SH, Lovibond PF (1995). Manual for the depression anxiety stress scales.

[38] Laugwitz B, Schrepp M, Held T. Konstruktion eines Fragebogens zur Messung der User Experience von Softwareprodukten. In: Paul AMHH, editor. Mensch & Computer 2006 – Mensch und Computer im Strukturwandel (S.125–134). München, Germany: Oldenbourg Verlag; 2006.

[39] Attkisson CC, Zwick R (1982). The client satisfaction questionnaire. Psychometric properties and correlations with service utilization and psychotherapy outcome. Eval Program Plann.

[40] Becker A, Seitz R, Jacobi E, Leidl R (2001). Kostenmessung durch Patienten- befragung: Pilotstudie zu einem Kostenwochenbuch. Rehabil..

[41] Schwarz D (2014). Die Zufriedenheit zuweisender Ärzte mit einem Programm zur angeleiteten Selbsthilfe für Eltern von Kindern mit ADHS.

[42] Borm GF, Fransen J, Lemmens WA (2007). A simple sample size formula for analysis of covariance in randomized clinical trials. J Clin Epidemiol.

